# Ovarian low and high grade serous carcinomas: hidden divergent features in the tumor microenvironment

**DOI:** 10.18632/oncotarget.10797

**Published:** 2016-07-23

**Authors:** Alessandra Ciucci, Gian Franco Zannoni, Marianna Buttarelli, Enrica Martinelli, Floriana Mascilini, Marco Petrillo, Gabriella Ferrandina, Giovanni Scambia, Daniela Gallo

**Affiliations:** ^1^ Unit of Translational Medicine for Women and Children Health, Department of Obstetrics and Gynecology, Catholic University of the Sacred Heart, Rome, Italy; ^2^ Department of Pathology, Catholic University of the Sacred Heart, Rome, Italy; ^3^ Department of Obstetrics and Gynecology, Catholic University of the Sacred Heart, Rome, Italy; ^4^ Department of Medicine and Health Sciences, University of Molise, Campobasso, Italy

**Keywords:** ovary, tumor-associated macrophages, TAM, M1, M2

## Abstract

Only recently low-grade serous carcinoma (LGSOC) of the ovary has been recognized as a disease entity distinct from the more common high-grade serous carcinoma (HGSOC), with significant differences in pathogenesis and clinical and pathologic features. The present study aimed at evaluating whether the different natural histories and patterns of response to therapy demonstrated for LGSOC and HGSOC, along with a diverse genomic landscape, may also reside in the supporting tumor stroma, specifically in the state of differentiation and activation of tumor associated macrophages (TAMs). TAMs play complex roles in tumorigenesis since they are believed to possess both tumor rejecting (M1 macrophages) and tumor promoting (M2 macrophages) activities. Here we showed that, when compared to HGSOC (*n* = 55), LGSOC patients (*n* = 25) exhibited lower density of tumor-infiltrating CD68+ macrophage, along with an attenuated M2-skewed (CD163+) phenotype. Accordingly, assessment of intratumoral vascularization and of matrix metalloproteinase 9 expression (a key protein involved in tumor invasion and metastasis) revealed lower expression in LGSOC compared to HGSOC patients, in line with emerging evidence supporting a role for TAMs in all aspects of tumor initiation, growth, and development. In conclusion, results from the present study demonstrate that microenvironmental factors contribute greatly to determine clinical and pathological features that differentiate low and high grade serous ovarian carcinomas. This understanding may increase possibilities and opportunities to improve disease control and design new therapeutic strategies.

## INTRODUCTION

Ovarian cancer is the most deadly gynecologic malignancy [1]. This insidious disease is often diagnosed in an advanced stage, develops rapidly and therefore has a poor prognosis. Over 90% of ovarian malignancies are categorized as epithelial ovarian cancers, and currently five main types are identified: high-grade serous, low-grade serous, mucinous, endometrioid, and clear-cell carcinoma. Low-grade and high-grade serous ovarian cancers actually comprise ∼70% of all epithelial ovarian tumors and account for the majority of deaths. In line with the evidence that ovarian cancer represents a group of distinct entities with distinct types of carcinogenesis, it is now widely accepted that low-grade and high-grade serous tumors are essentially distinct diseases, exhibiting distinct genetic alterations, molecular patterns and clinical behaviors. Specifically, the former develop from well-recognized precursors and behave in an indolent fashion, are characterized by specific mutations, including *KRAS*, *BRAF* and *ERBB2* and are relatively genetically stable. In contrast, HGSOCs are suggested to be more aggressive, found at advanced stage, and genetically highly unstable. The majority have TP53 mutations, but rarely harbor the mutations detected in the low-grade serous tumors [2].

Overlaying this complexity is the contribution of supporting cells, and the tumor microenvironment is now increasingly recognized to play an important role in epithelial ovarian cancer [3]. The microenvironment of solid tumors is indeed characterized by a reactive stroma with a plenty of inflammatory cells, dysregulated vessels and proteolytic enzymes. Inflammatory infiltrates include a rich supply of macrophages, which are recruited by tumor cells through their secretion of chemokines [3, 4]. Actually, tumor cells and macrophages engage in a bidirectional interaction through the exchange of soluble mediators, which influence cell behavior and phenotype [4]. Macrophages constitute an extremely heterogeneous population which differentiate into distinct types, schematically identified as M1 (or classically activated) and M2 (or alternatively activated) [5]. “Classically activated” M1 macrophages contribute to tumor rejection through type 1 cytokine production and antigen presentation, whereas “alternatively activated” M2 macrophages enhance angiogenesis and remodeling, through type 2 cytokine production. It is now generally accepted that tumor-associated macrophages (TAM) most closely resemble M2-polarized cells, creating an immunosuppressive microenvironment and finally promoting tumor invasion, angiogenesis, and metastasis [5]. If the tumor is small, TAMs derived from the surrounding tissue macrophages represent the majority of TAMs, while as the cancer mass rises and an intratumoral vascular network forms, monocyte-derived TAMs turn out to be the main source of TAMs [6]. In the primary tumor, TAMs create an immunosuppressive microenvironment promoting angiogenesis, tumor invasion, motility and intravasation. During metastasis, macrophages prime the pre-metastatic site and promote tumor cell extravasation, survival and persistent growth [7]. Previous studies aimed at characterizing TAMs in ovarian cancer demonstrated that they most closely resemble M2-polarized macrophages and express M2 markers such as CD163, CD204, CD206 (Mannose Receptor), and IL-10 [4]. Moreover, co-culture of human macrophages with ovarian cancer cell lines was associated with the polarization to the M2 phenotype [8].

Despite an increasing amount of evidence is emerging to suggest that TAMs display a unique activation profile in ovarian tumors, many questions still remain, and, among these, the contribution of this immune cell type in each of the histopathological ovarian cancer subtypes is likely to be really complex and requires investigations. The present study aimed at evaluating whether the different natural histories and patterns of response to therapy demonstrated for LGSOCs and HGSOCs, along with a diverse genomic landscape, may also reside in the supporting tumor stroma, specifically in the state of differentiation and activation of TAMs, which in turn, may promote a different tumor development and spread.

## RESULTS

The study population included 25 LGSOC and 55 HGSOC patients. Patient characteristics are summarized in Tables 1 and 2. The mean age of patients with LGSOC was significantly lower than in the HGSOC group (49.8 ± 3.0, and 56.8 ± 1.5, respectively, mean ± SEM, *p* = 0.03, Table 1), in keeping with literature data [9, 10]. In addition, HGSOC patients were more likely to have advanced-stage disease, compared to LGSOC ones (*p* = 0.01, Table 1). Follow-up information was available for all cases, with LGSOC and HGSOC patients having mean follow-up times of 51 (9–180) and 47 (7–140) months, respectively, from the date of surgery (Table 2). On follow up, most of LGSOC patients were alive without evidence of recurrence, while the majority of HGSOC eventually recurred and died of disease (Table 2).

**Table 1:** Clinicopathological features of the overall series

Characteristics	LGSOC No. of patients (%)	HGSOC No. of patients (%)	*p* values
**All cases**	25	55	
**Mean Age, years (± SEM)**	49.8 ± 3.0	56.8 ± 1.5	0.03
**Type of primary surgery**			
Cytoreduction	25	55	–
**Residual tumor after primary surgery (cm)**			
0	22	36	0.06#
< 1	3	14	
> 1	0	5	
**FIGO Stage**			
I/II	10 (40)	8 (15)	0.01
III/IV	15 (60)	47 (85)	
**Primary chemotherapy**			
None	3 (12)	1 (1.8)	0.08§
Platinum/paclitaxel	17 (68.0)	48 (87.3)	
Platinum-based	4 (16.0)	5 (9.1)	
Other	1 (4)	1 (1.8)	

*P* values were calculated using the Fisher's exact test;

# No residual tumor versus any extent of residual disease;

§ No chemotherapy *versus* any type of chemotherapy.

**Table 2:** Clinical outcome in the overall series

Characteristics	LGSOC No. of patients (%)	HGSOC No. of patients (%)
**All cases**	25	55
**Mean Follow up, months (range)**	51 (9–180)	47 (7–140)
**Clinical status**		
Alive	23 (96)	22 (40)
Alive NED	19 (76)	14 (26)
Dead	2 (4)	33 (60)
Recurrent (total)	5 (20)	41 (68)
Stage I/II	1	1
Stage III/IV	4	40

NED = No evidence of disease.

### Total and M2-polarized macrophage infiltration in LGSOCs and HGSOCs

**Figure 1:**
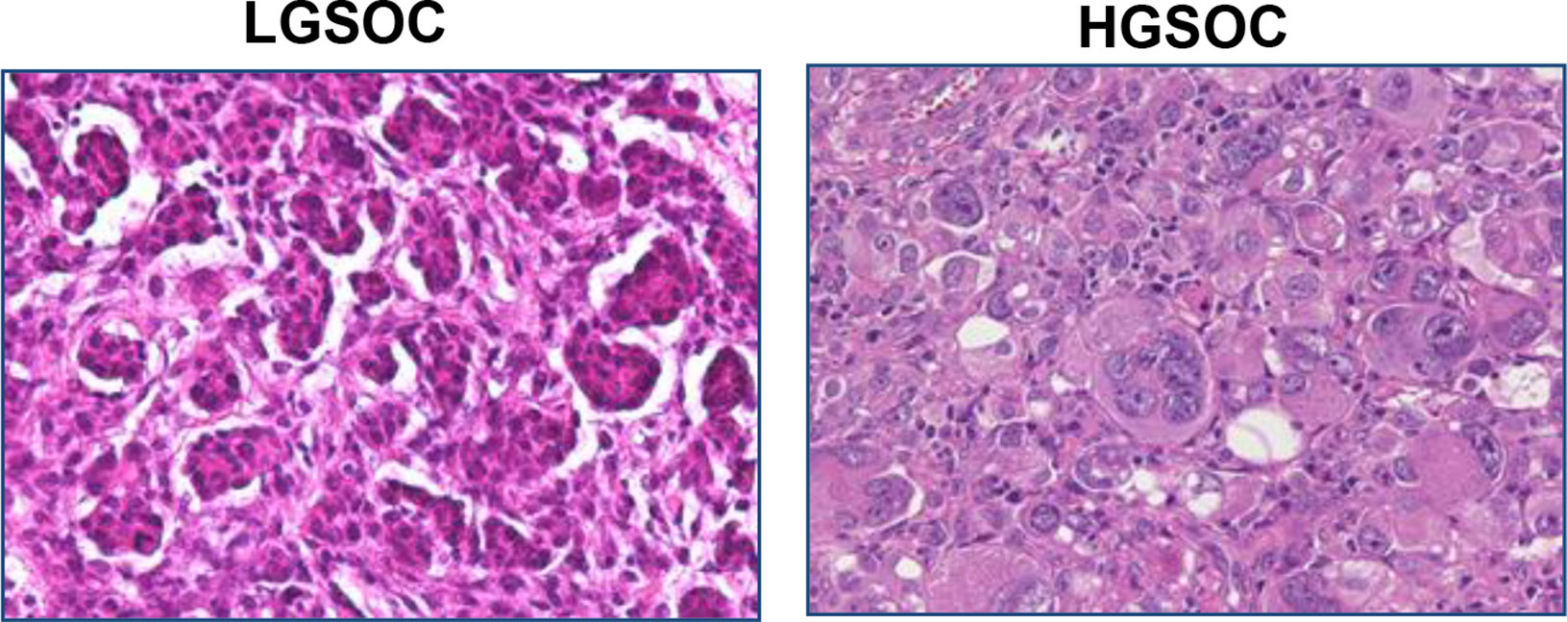
Histological features of LGSOCs and HGSOCs Low-grade serous carcinoma of the ovary is characterized by relative uniformity of the cells and up to 12 mitoses per 10 high-power fields. High-grade serous carcinoma of the ovary is characterized by pleomorphism, marked nuclear atypia and > 12 mitoses per 10 high-power fields.

**Figure 2:**
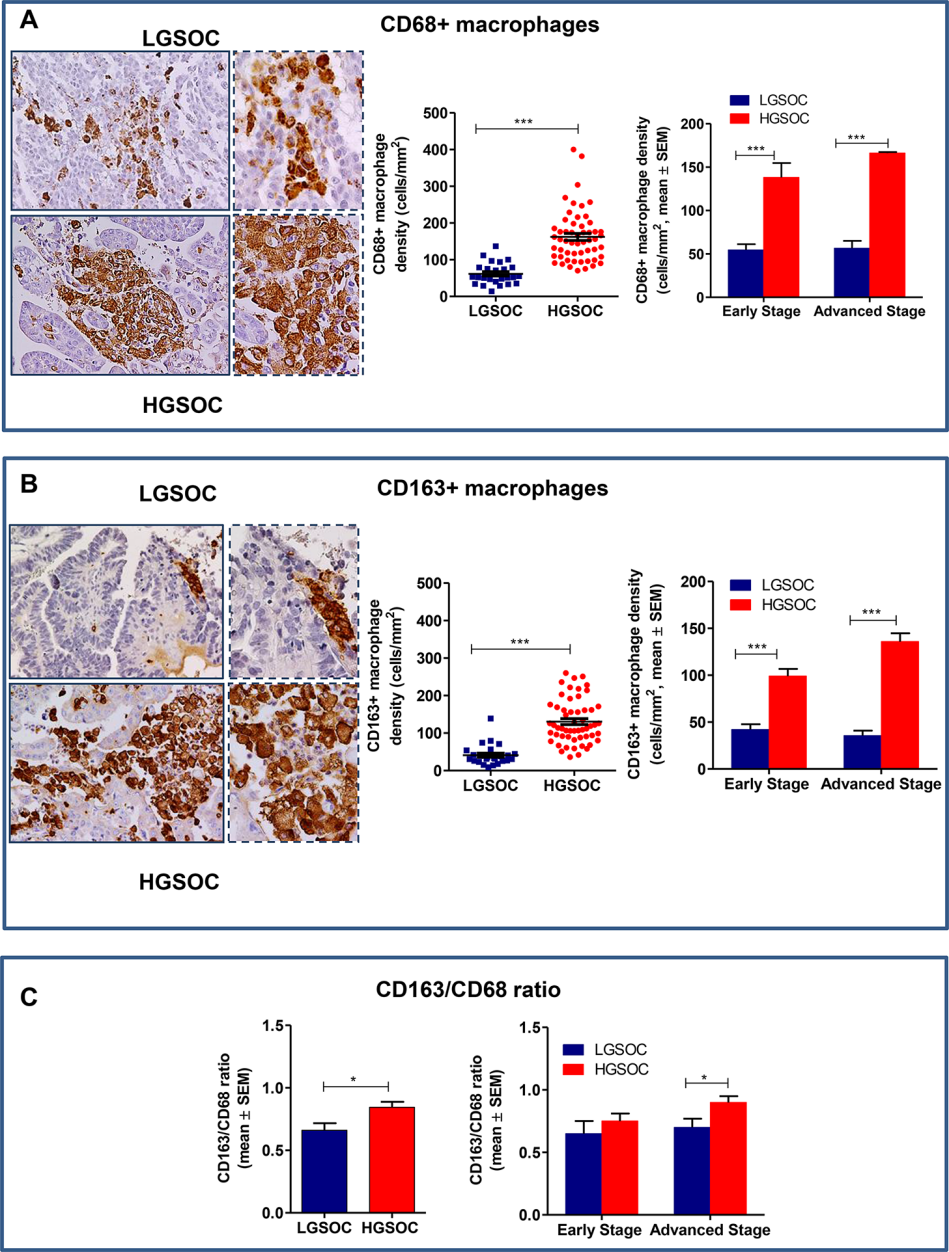
Densities of tumor associated macrophages in LGSOC and HGSOC tissue specimens (**A**) Representative pictures for immunohistochemical staining of CD68+ macrophages in clinical samples of LGSOC and HGSOC. Magnification 20× and 40×. Scatter plot shows all data points and mean ± SEM for the entire set of patients (*n* = 25 and *n* = 55, for LGSOCs and HGSOCs respectively, ***p < 0.0001). Bar graphs depict data (mean ± SEM) following stratification per stage (Early stage, *n* = 10 and *n* = 8; Advanced Stage *n* = 15 and *n* = 47, for LGSOCs and HGSOCs respectively, ***p < 0.0001). (**B**) Representative pictures for immunohistochemical staining of CD163+ macrophages in clinical samples of LGSOC and HGSOC. Magnification 20× and 40×. Scatter plot shows all data points and mean ± SEM for the entire set of patients (see above for sample sizes, ***p < 0.0001). Bar graphs depict data (mean ± SEM) following stratification per stage (see above for sample sizes, ***p < 0.0001). (**C**) Bar charts showing the CD163/CD68 ratio (mean ± SEM) in the entire population (see above for sample sizes, *p = 0.02) and after stratification per stage (see above for sample sizes, *p = 0.049).

To establish total and M2-polarized macrophage infiltration in cancer tissues, surgically collected human LGSOCs and HGSOCs (Figure 1) were immunohistochemically stained for CD68 and CD163 (Figure 2). Indeed, CD68 and CD163 are both used to identify macrophages in tissue sections, but while CD68 is commonly used as a pan-macrophage marker, CD163 is regarded as a highly specific marker for M2-polarized macrophages in several human tumors, including ovarian cancer [11–19].

Results obtained showed that total macrophage infiltration (CD68+) was significantly lower in LGSOC compared to HGSOC patients (61 ± 5.9 and 162 ± 9.4 cells/mm^2^, respectively, mean ± SEM, *p* < 0.0001, Figure 2A). Likewise, the density of CD163+ macrophages was significantly lower in LGSOC compared to HGSOC cases (41 ± 5.4 and 130 ± 7.9 cells/mm^2^, respectively, mean ± SEM, *p* < 0.0001, Figure 2B). Data stratification per stage and subsequent comparison, confirmed significant differences between the two histotypes, independently of disease stage (Figure 2A and 2B). We also calculated the CD163/CD68 ratio (i.e. the number of M2 macrophages within the total macrophage count) for the two populations, results showing that LGSOC patients exhibited an attenuated M2-skewed phenotype compared to HGSOC ones (0.6 ± 0.05 and 0.8 ± 0.04, respectively, mean ± SEM, *p* = 0.02, Figure 2C). Statistical analysis after stratification per stage confirmed this trend only in women with advanced disease (*p* = 0.049, Figure 2C).

### Microvessel density in LGSOCs and HGSOCs

**Figure 3:**
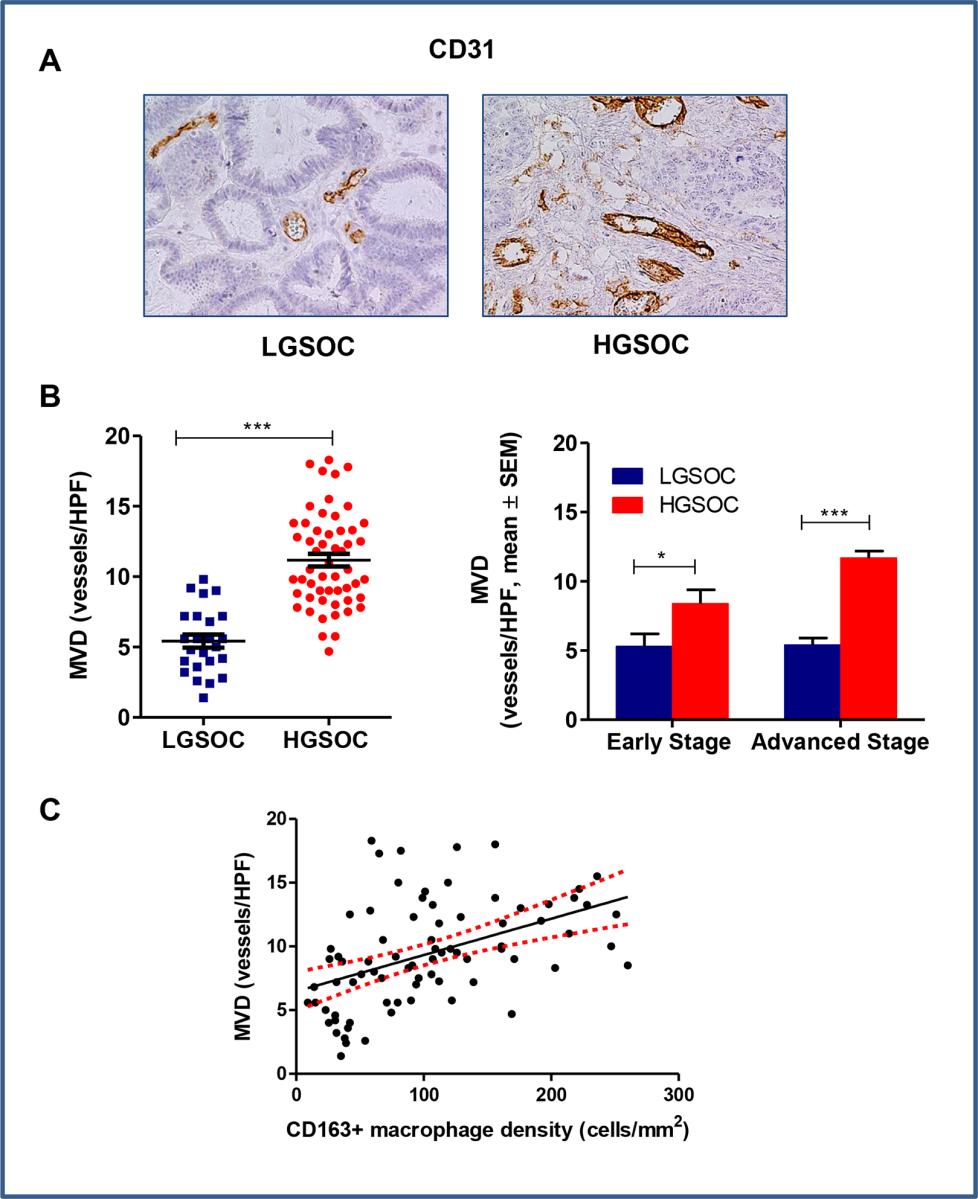
Tumor-associated angiogenesis in LGSOC and HGSOC tissue specimens (**A**) Representative pictures for immunohistochemical staining of CD31 in clinical samples of LGSOC and HGSOC. Magnification 20×. (**B**) Scatter plot shows MVD (Microvessel Density, vessels/HPF) values and mean ± SEM for the entire set of patients (see legend to Figure 2 for sample sizes, ***p < 0.0001). Bar graphs depict data (mean ± SEM) following stratification per stage (see legend to Figure 2 for sample sizes, *p = 0.04, ***p < 0.0001). (**C**) The Spearman rank correlation showed a significant positive correlation between MVD and CD163+ macrophages density (cells/mm^2^) (*n* = 80, *p* < 0.0001).

Clinical evidence shows a correlation between local macrophage density and areas of intense angiogenesis defined by the presence of microvessels, suggesting that the angiogenic switch in tumors depends on macrophage infiltration [20]. We thus assessed the microvessel density (MVD) in tumors using CD31, a specific and sensitive endothelial marker for formalin-fixed paraffin-embedded tissues [21]. LGSOC patients had significantly lower microvessel densities compared to HGSOCs, these latter showing a dense network of vessels with multiple branching (MVD = 5.4 ± 0.5 and 11.2 ± 0.5 vessels/HPF, respectively, mean ± SEM, *p* < 0.0001; Figure 3A and 3B). Data stratification per stage and subsequent comparison, confirmed significant differences between the two histotypes, independently of disease stage (*p* = 0.04 and *p* < 0.0001 for early and advanced stage patients, respectively, Figure 3B). Notably, the Spearman rank correlation showed a significant positive correlation between MVD and CD163+ macrophage density (*r* = 0.5 *p* < 0.0001) (Figure 3C).

### MMP-9 expression in LGSOCs and HGSOCs

**Figure 4:**
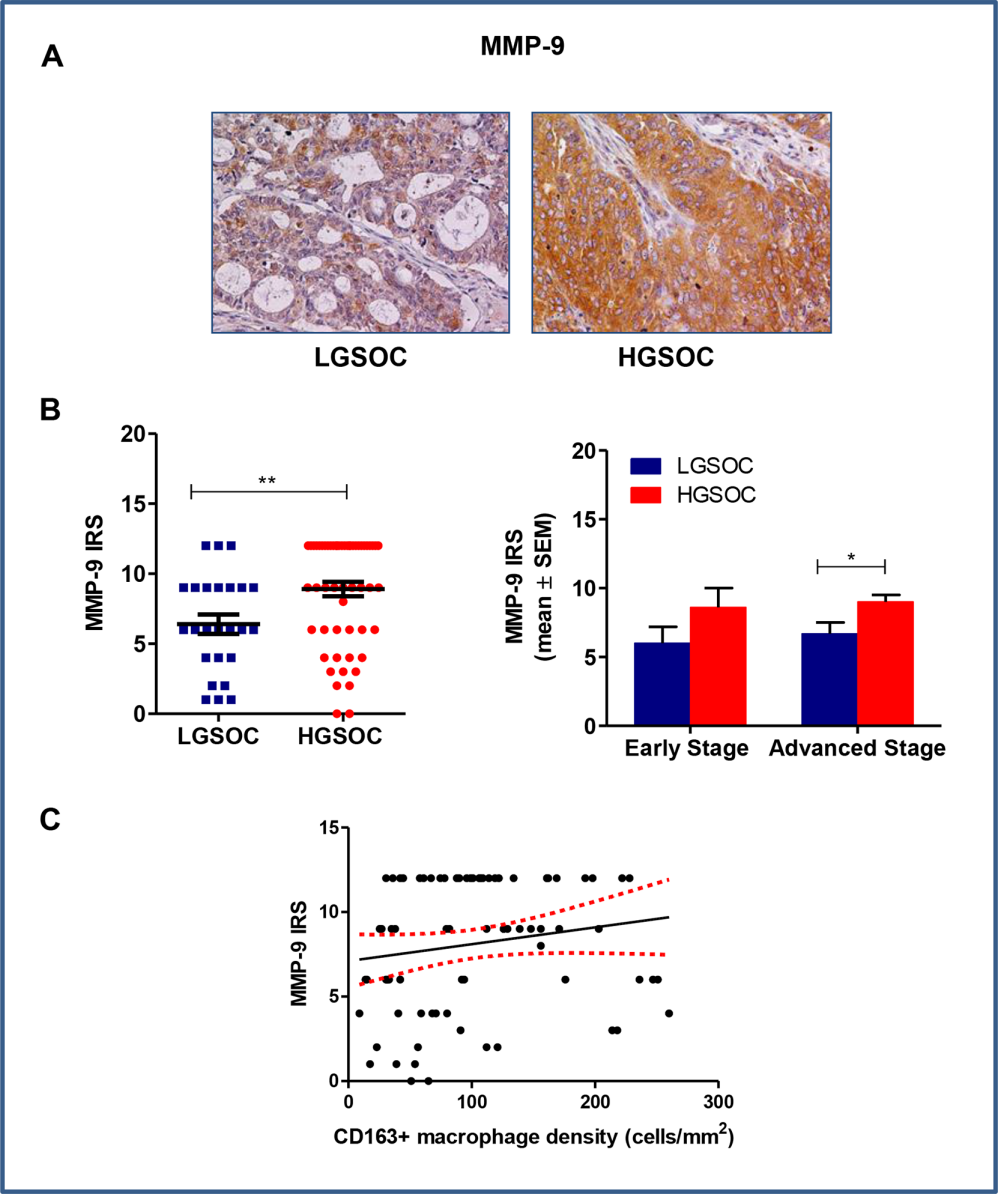
Matrix metalloproteinases 9 (MMP-9) expression in LGSOC and HGSOC tissue specimens (**A**) Representative pictures for immunohistochemical staining of MMP-9 in clinical samples of LGSOC and HGSOC. Magnification 20×. (**B**) Scatter plot shows MMP-9 IRS (Immunoreactive receptor score) values and mean ± SEM for the entire set of patients (see legend to Figure 2 for sample sizes, **p = 0.006). Bar graphs depict data (mean ± SEM) following stratification per stage (see legend to Figure 2 for sample sizes, *p = 0.04). (**C**) The Spearman rank correlation showed a significant positive correlation between MMP-9 IRS and CD163+ macrophages density (cells/mm^2^) (*n* = 80, *p = 0.04).

Matrix metalloproteinase-9 (MMP-9) is a zinc-dependent peptidase, belonging to the gelatinase subfamily of MMPs. It is excreted as an inactive pro-enzyme that undergoes activation upon cleavage by different types of extracellular proteases, and mediates extracellular matrix (ECM) degradation, thus playing a key role in tumor invasion and metastasis, and in tumor-induced angiogenesis. In some tumors, TAM appeared to be a major source of MMP-9 [22]. On the basis of these findings, we used immunohistochemistry to assess MMP-9 expression in our series of high- and low-grade serous ovarian cancers (Figure 4A and 4B). Data obtained demonstrated that LGSOC patients expressed significantly lower MMP-9 protein than HGSOC ones (IRS 6.4 ± 0.6 and 8.9 ± 0.5 for LGSOCs and HGSOCs, respectively, mean ± SEM, *p* = 0.006). After stratification per stage and subsequent comparison, differences in MMP-9 expression between the two histotypes remained significant for stage III-IV only (IRS 6.6 ± 0.8 and 9.0 ± 0.6 for LGSOC and HGSOC, respectively, mean ± SEM, *p* = 0.04), while no significant changes were found at lower stages (Figure 4B). A Spearman correlation analysis showed a significant positive correlation between MMP-9 and CD163+ macrophages density (*r* = 0.2, *p* = 0.04) (Figure 4C).

### E-cadherin expression in LGSOCs and HGSOCs

**Figure 5:**
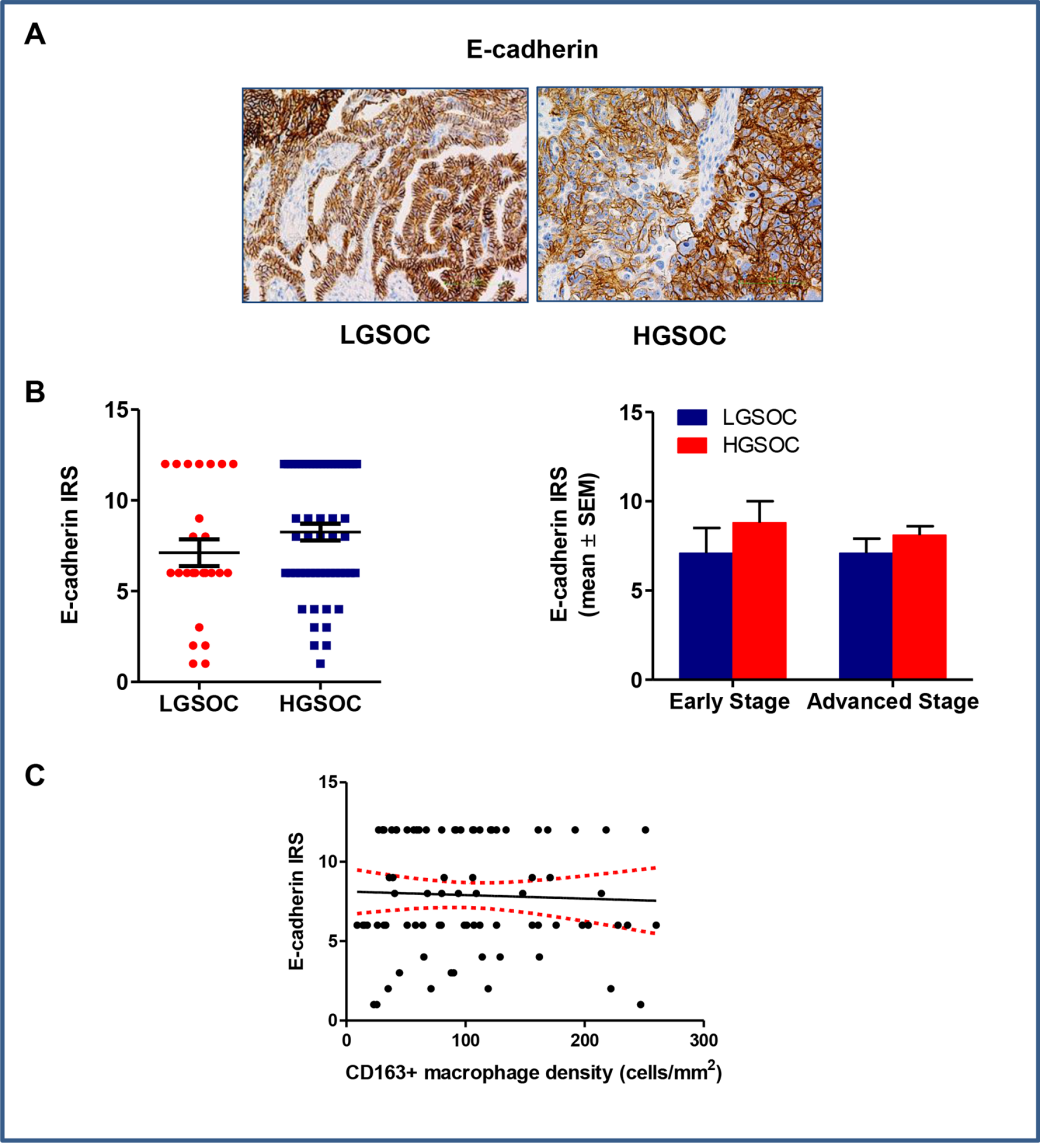
E-cadherin expression in LGSOC and HGSOC tissue specimens (**A**) Representative images for immunohistochemical staining of E-cadherin in clinical samples of LGSOC and HGSOC. Magnification 20×. (**B**) Scatter plot shows E-cadherin IRS (Immunoreactive receptor score) values and mean ± SEM for the entire set of patients (see legend to Figure 2 for sample sizes). Bar graphs depict data (mean ± SEM) following stratification per stage (see legend to Figure 2 for sample sizes). (**C**) There was no correlation between expression of E-cadherin and CD163+ macrophages density (cells/mm2) (*n* = 80), as showed by the Spearman rank analysis.

Epithelial to mesenchymal transition (EMT) plays a fundamental role in tumor progression and metastasis formation, and accumulated evidences have demonstrated that TAMs plays critical role in the regulation of EMT in cancer [23]. To verify this hypothesis, we chose membranous E-cadherin as epithelial marker and evaluated its expression in our series of low- and high-grade serous ovarian cancers (Figure 5A and 5B). Results obtained did not show any significant differences in protein expression between LGSOC and HGSOC samples (IRS 7.1 ± 0.7 and 8.1 ± 0.5 for LGSOC and HGSOC, respectively, mean ± SEM). Paired comparison after data stratification per stage confirmed the similar distribution in E-cadherin levels between the two series examined (Figure 5B). As expected on the basis of these results, Spearman analysis did not show any correlation between CD163+ macrophages density and E-cadherin expression (*r* = −0.001, *p* = 0.98, Figure 5C).

## DISCUSSION

Significant clinical, pathologic, and pathogenesis differences have been described between LGSOC and HGSOC, although most research on the diversity of these two cancers has been focused on the impact of cancer cell biology [9]. However, cancers develop in composite tissue environments (that they depend upon for growth, invasion and metastasis) consisting of matrix components, inflammatory cells, and stromal cells. Therefore, in this study we sought to investigate whether, besides tumor cell-intrinsic factors, microenvironmental factors can contribute to determine clinical and pathological features that differentiate low and high grade serous ovarian carcinomas. Notably, we show here, for the first time, that LGSOC and HGSOC exhibit striking differences in tumor-associated macrophage infiltration and, more importantly, in their activation profile, findings which were in turn related to different tumor vascularization and expression of key proteins involved in tumor growth and metastases.

Indeed, we found that, when compared to HGSOC, LGSOC patients showed a lower density of tumor-infiltrating CD68+ macrophage along with an attenuated M2-skewed (CD163+) phenotype. Notably, this trend was confirmed when patients with early- and late-stage disease were analyzed separately, this suggesting that the subpopulations of EOC cells composing the diverse tumors can differentially affect the process of immune cell infiltration and differentiation. In line with these results and with the notion that low-grade have better outcomes than high-grade tumors, literature data strongly support a role of TAMs as prognostic factors in ovarian cancer [reviewed in 4]. As far as we know, results described here are the first showing differences in TAM distribution patterns between low- and high-grade serous ovarian cancers. In fact, previous studies in this area did not analyze separately LGSOC and HGSOC, but considered the serous histotype on the whole, also producing contradictory results. In detail, while some Authors demonstrated significant differences in the TAM infiltration according to cancer histotype (TAM most frequently infiltrating serous and mucinous, compared to other histotypes) [24], others did not find any relationship between the density of CD68/CD163-positive cells and ovarian cancer histological type [18]. Moreover, our results also showed that there were no significant changes in the overall TAM profile when comparing, within the same subtype, early- and advanced-stage disease, this suggesting that distinct tumor microenvironments support the growth and development of low- and high-grade serous ovarian cancer independently on tumor stage.

Tumor-associated macrophages have been found to promote ovarian tumor by employing several different strategies, including promotion of angiogenesis. Indeed, TAMs preferentially accumulate in hypoxic and necrotic regions within the tumors and cooperate with tumor cells to boost the angiogenic switch [25]. Several recent studies have indeed demonstrated that not only TAMs function as major producers of a panel of pro-angiogenic factors (i.e. growth factors, cytokines, and chemokines) in malignant tumors, but they also induce a pro-angiogenic program in tumor cells [22]. In keeping with these literature data, we found a strong association between intra-tumor TAM density and microvessel density, so that CD31 expression closely paralleled density of tumor-infiltrating CD163+ macrophage in low and high-grade serous ovarian cancer. Notably, molecular support to our observation in clinical specimens, is provided by data from Wang and colleagues [26], showing, *in vitro* models, that the interaction of ovarian cancer cells and TAMs enhances the ability of endothelial cells to promote the progression of ovarian cancer.

Once the barrier of the angiogenic switch has been overcome, tumors rapidly become invasive. For metastasis to occur, a crucial step is the destruction of biological barriers, such as the basement membrane, which requires activation of proteolytic enzymes. Key proteins in this process include the matrix metalloproteinases (MMPs) [27] and recent literature data have provided evidence of a strong association between TAMs and MMPs levels [22], showing that is through the production of proteolytic enzymes and MMPs that TAMs reorganize the extracellular matrix and degrade the basement membrane [28]. Actually, Spiller and colleagues [29] demonstrated that M2c macrophages (distinguished by expression of the scavenger receptor CD163) secrete the highest levels of MMP-9. Our results fit this mechanism well, since we found that there is a positive correlation between CD163+ macrophages and MMP-9 tumor levels, as demonstrated by Spearman analysis. Hence, LGSOC patients showing an attenuated M2-skewed phenotype (CD163+) compared to HGSOC, also expressed significantly lower MMP-9 expression in tumor samples.

Recent studies have postulated that TAMs triggers EMT through regulation of different signaling pathways in cancer [23, 30], with E-cadherin showing a negative correlation with CD68+ macrophage density [30]. However, unlike most carcinomas that dedifferentiate during neoplastic progression with loss of epithelial E-cadherin, ovarian carcinomas undergo transition to a more epithelial phenotype, early in tumor progression, with increased E-cadherin expression. Subsequent reacquisition of mesenchymal features is observed in late-stage tumors, and loss of E-cadherin expression or function may occur in ovarian cancer progression [reviewed in 31]. We thus assessed E-cadherin expression in our series of low- and high-grade serous ovarian cancers, to verify whether any differences occurred in the two series and, if so, whether these differences had any relationship to TAM density. Data obtained showed a similar distribution in E-cadherin levels between the two series examined, and protein expression was not correlated to density of tumor-infiltrating CD163+ macrophage. Our data confirm previous literature reporting that ovarian epithelial cancers express high levels of E-cadherin regardless of tumor type, stage of malignancy, or stage of differentiation [32, 33], with a strong positivity in HGSOC described in more than 85% of cases [34]. However, some discrepancies exist since other authors reported higher E-cadherin expression in LGSOC compared to HGSOC [35, 36]. It is interesting to note, however, that mechanistic studies proved that in ovarian cancer cells, E-cadherin may serve not only as an intercellular adhesion molecule, but also as an upstream regulator that triggers downstream kinase activation, this explaining why E-cadherin is always expressed during ovarian tumor development and progression [33, 37]. Additional studies on a larger number of cases are certainly needed to clarify these unresolved issues.

In conclusion, results from the present study give a substantial contribution in the definition of macrophage subpopulations in low- and high-grade serous ovarian cancer, this aligning with the drive to understand the tumor microenvironment and cancer cell biology to improve disease control.

Notably, in spite of differences in histology and clinical outcomes, patients with LGSOC and HGSOC are currently treated with the same treatments, which are not that effective in LGSOC [38]. Thus, new therapeutic strategies and novel molecular targets are needed to improve the outcome of this patient cohort, and TAM might represent an attractive target of novel biological therapies. As recently reviewed by Williams and colleagues [28], macrophage-targeted intervention strategies may actually represent a cornerstone in cancer treatment, particularly in association with conventional or novel ovarian cancer interventions.

## MATERIALS AND METHODS

### Patients

This retrospective study included specimens collected for clinical purposes between the years 2002 and 2014 at the Gynecologic Oncology Unit, Catholic University of Rome, Italy. Histologic grading of ovarian carcinomas was revised according to the 2014 WHO Classification of Tumors of the Female Genital Tract [39]. A total of 25 LGSOC and 55 HGSOC tissue samples were included in the study. In our Institution a written informed consent is routinely requested from patients for collection of their clinical data, as well as paraffin embedded sections for research use. Clinical information was obtained from the existing medical records in accord with institutional guidelines. All data were managed using anonymous numerical codes.

### Immunohistochemistry

Immunohistochemical studies were performed on formalin-fixed, paraffin-embedded sections as previously described [19, 40], or in a Dako AutoStainer (Dako, Carpinteria, CA). Antibodies used include: anti-CD68 (clone PG-M1, Dako, dilution 1:100); anti-CD163 (clone10D6, Biocare Medical, Concord, CA, USA, dilution 1:50); anti-CD31 (clone JC70A, Dako, ready-to use); anti-MMP-9 (clone Ab-2, Oncogene Research Products Cambridge, MA, dilution 1:50); and anti E-cadherin (Dako, clone NCH-38, ready to use).

### Evaluation of immunohistochemical staining

Tumor-associated macrophage (TAM) densities were assessed by counting the number of intratumoral macrophages with positive staining for the phenotype marker(s) in four representative 400× high-power fields (total tumor surface: 1 mm^2^). Macrophage density was expressed as cells/mm^2^. For the quantitative analysis of microvessel density, CD31-positive intratumoral microvessels were counted blindly under a microscope field (×400 objective magnification, high-power field area = 0.24 mm^2^). A minimum of 4 tumor areas per section were evaluated and the microvascular density (MVD) was then expressed as mean number of vessel per high-power field (MVD, vessels/HPF). For MMP-9, the intensity of cytoplasmatic staining and the percentage of immunoreactive cells to total tumor cells were evaluated. The extent of expression was scored 0 for no staining, 1 = 1–10%, 2 = 11–33%, 3 = 34–66%, 4 = 67–100%. A similar semiquantitative scale of 0, +, ++, or +++ was used to assess the intensity of staining. The two values obtained were multiplied to calculate an immunoreactive score (IRS, maximum value 12) [41]. The E-cadherin immunoreactivity was recognized as a membrane staining signal. The immunoreactive score was calculated as described above for MMP-9. Immunohistochemical assessment was carried out by two investigators blinded to groups.

### Statistical analysis

Differences between groups in clinicopathological parameters were evaluated using the Fisher's exact test. All other data were analyzed for homogeneity of variance using an *F* test. If the variances were heterogeneous, log or reciprocal transformations were made in an attempt to stabilize the variances, followed by Student's *t*-test. If the variances remained heterogeneous, a non-parametric test such as the Mann–Whitney *U* test was used. Data are reported as mean ± SEM. *P* values are for two-sided tests; *p* values ≤ 0.05 were considered statistically significant. Analyses were performed using GraphPad Prism version 5.0 for Windows (GraphPad Software, San Diego, CA).
